# Predictors and outcomes of sustained, intermittent or never achieving remission in patients with recent onset inflammatory polyarthritis: results from the Norfolk Arthritis Register

**DOI:** 10.1093/rheumatology/kew210

**Published:** 2016-05-24

**Authors:** Michael J. Cook, Janet Diffin, Carlo A. Scirè, Mark Lunt, Alex J. MacGregor, Deborah P. M. Symmons, Suzanne M. M. Verstappen

**Affiliations:** ^1^Arthritis Research UK Centre for Epidemiology, Centre for Musculoskeletal Research, Institute for Inflammation and Repair, Manchester Academic Health Science Centre; ^2^School of Nursing, Midwifery & Social Work, The University of Manchester, Manchester, UK; ^3^Italian Society for Rheumatology, Epidemiology Unit, Milan, Italy; ^4^Norwich Medical School, University of East Anglia, Norwich; ^5^NIHR Manchester Musculoskeletal Biomedical Research Unit, Central Manchester University Hospitals NHS Foundation Trust, Manchester, UK

**Keywords:** inflammatory polyarthritis, rheumatoid arthritis, remission, predictors, disease progression

## Abstract

**Objectives.** Early remission is the current treatment strategy for patients with inflammatory polyarthritis (IP) and RA. Our objective was to identify baseline factors associated with achieving remission: sustained (SR), intermittent (IR) or never (NR) over a 5-year period in patients with early IP.

**Methods.** Clinical and demographic data of patients with IP recruited to the Norfolk Arthritis Register (NOAR) were obtained at baseline and years 1, 2, 3 and 5. Remission was defined as no tender or swollen joints (out of 51). Patients were classified as NR or PR, respectively, if they were in remission at: no assessment or ⩾3 consecutive assessments after baseline, and IR otherwise. Ordinal regression and a random effects model, respectively, were used to examine the association between baseline factors, remission group and HAQ scores over time.

**Results.** A total of 868 patients (66% female) were included. Of these, 54%, 34% and 12% achieved NR, IR and SR, respectively. In multivariate analysis, female sex (odds ratio, OR 0.47, 95% CI: 0.35, 0.63), higher tender joint count (OR = 0.94, 95% CI: 0.93, 0.96), higher HAQ (OR = 0.59, 95% CI: 0.48, 0.74), being obese (OR = 0.70, 95% CI: 0.50, 0.99), hypertensive (OR = 0.67, 95% CI: 0.50, 0.90) or depressed (OR = 0.74, 95% CI: 0.55, 1.00) at baseline were independent predictors of being in a lower remission group. IR and SR were associated with lower HAQ scores over time and lower DAS28 at year 5.

**Conclusion.** Women with higher tender joint count and disability at baseline, depression, obesity and hypertension were less likely to achieve remission. This information could help when stratifying patients for more aggressive therapy.

Rheumatology key messages

- Factors including comorbidity and obesity are significant predictors of remission in inflammatory polyarthritis.
- Predictors of remission may be useful for stratifying patients with inflammatory polyarthritis for more aggressive therapy.
- Patients with inflammatory polyarthritis who achieved remission had improved functional ability over a five-year follow-up.

## Introduction

Remission, in patients with inflammatory polyarthritis (IP) or its subset RA, can be defined as the absence of disease, with no detectable symptoms, signs or markers of inflammation [1]. The ultimate aim of treatment for RA is to achieve early and sustained remission [2]. In recent years, remission has become a more realistic target due to improved treatments and more aggressive regimes being administered earlier in the disease course [3]. However, in published studies remission rates vary considerably for patients with IP, and it is difficult to identify at disease onset which patients will ever achieve remission or achieve sustained remission [4, 5].

Remission has the greatest beneficial impact on long-term functional disability, work disability and radiographic damage if it is sustained over a period of time [6–9]. In a systematic review including 18 papers published prior to November 2008, Katchamart *et al.* [5] reported that male sex, young age, late onset RA (age >65 years), shorter disease duration, non-smoker, low baseline disease activity, lower functional impairment, absence of RF and anti-CCP, early treatment with DMARD combinations and the concurrent use of DMARDs with TNF inhibitors were predictors of remission at the end of the study in patients with RA. However, most previous studies of predictors of remission have assessed remission only at the end point of the study over a relatively short follow-up period of between 1 and 3 years, and often have not considered the impact of comorbidities [10–15].

The primary aim of this study was to identify factors measured at an early stage of disease that are associated with sustained (SR), intermittent (IR) or never achieving remission (NR) within the Norfolk Arthritis Register (NOAR) over a 5-year follow-up. In addition, we evaluated the association between remission group and functional disability over time; and between remission group and DAS28-CRP at the fifth year anniversary.

## Methods

### Patients and setting

Patients recruited to NOAR, a primary-care-based inception cohort of patients with IP in Norfolk, UK, between 2000 and 2008 were included in this study [16]. The notification criteria were age over 16 years at symptom onset, and swelling of at least two joints that has lasted for at least 4 weeks. Patients were either referred by their general practitioner or rheumatologist and were treated in line with current national guidelines. Patients who were later diagnosed with a condition other than IP, RA or PsA, were excluded. We only included patients with symptom duration of less than 2 years at baseline. This study was given approval by the Norwich Local Research Ethics Committee. All patients provided written informed consent.

### Demographic and clinical assessments

At baseline and at 1, 2, 3 and 5 years after baseline, patients were assessed by a research nurse. Information collected included date of birth, sex, date of symptom onset, smoking status (current, ex- or never), height and weight (to compute BMI kg/m^2^), 51 swollen and tender joint count, DMARD and other medication use. Patients were classified as obese if they had a BMI ⩾ 30 kg/m^2^. The British version of the HAQ was completed by the patient [17]. Self-reported physician-diagnosed comorbidities were recorded by the research nurse, together with year of diagnoses. Comorbidities were selected from a list of predefined conditions [angina, hypertension, heart attack, heart failure, stroke, transient ischaemic attack, diabetes, stomach ulcer, liver disease, kidney failure, cancer (except skin cancer), psoriasis, depression, glaucoma] or recorded as free text. All comorbidity data were coded according to the International Statistical Classification of Diseases and Related Health Problems 10th Revision [18]. Blood samples were collected at baseline and at the fifth year assessment and stored at −80°C. Subsequently, blood samples were tested for RF (by latex method, positive at a titre of ⩾1:40), anti-CCP2 antibodies (by Axis-Shield Diastat kit, positive at ⩾5 U/ml) and CRP (by end point immunoturbidimetric agglutination method, in milligram per litre). The three-component 28-joint DAS28-CRP [19] was calculated at baseline and year 5. The ACR/EULAR 2010 classification criteria for RA were applied retrospectively using data collected at baseline [20]. Patients who died during follow-up (n = 29) or who had joint count data missing at one or more follow-up visits (n = 267) were excluded from the analysis (see supplementary Fig. S1, available at *Rheumatology* Online).

### Definition of remission

Remission was defined as no tender or swollen joints on examination of 51 joints. This definition provides a more stringent definition of remission than using a count of fewer joints and has previously been shown to be a better predictor of functional disability and mortality [21]. These criteria were applied at the first, second, third and fifth year assessments. Patients were classified as never achieving remission (NR) if they were not in remission at any anniversary, intermittently achieving remission (IR) if they were in remission at least once but ⩽2 consecutive assessments, and having sustained remission (SR) if they were in remission at ⩾3 consecutive assessments.

### Statistical analyses

The relationships between baseline variables and the three remission groups were analysed using ordinal logistic regression. Remission groups were ordered, with NR being the lowest group and SR being the highest group. The remission group was modelled as the dependent variable in three separate models: univariate; including age, sex and baseline oral steroid use as covariates; and including covariates selected by a forward stepwise variable selection procedure, with a significance value of <0.1 as the cut-off to be included in the model. All variables, apart from anti-CCP positivity (test for proportional odds, P = 0.01), satisfied the proportional odds condition of ordinal regression. For anti-CCP, multinomial regression analysis was used, and odds ratios for IR and SR are reported separately, with NR as the referent group. Separate ordinal regression analyses were performed: with no data imputed and with missing values imputed using Multiple Imputation by Chained Equations, with 20 imputations. The resulting models were compared. The random number generator seed was set in the multiple imputation process to ensure reproducible results. In the models that included imputed values, time from symptom onset to DMARD start date was replaced with the follow-up duration (5 years) for patients who did not receive DMARD treatment during follow-up. A dummy binary variable indicating whether each patient had received DMARD treatment during follow-up was considered in combination with the time between symptom onset and DMARD treatment in the stepwise variable selection process; both the dummy variable and the time from symptom onset to DMARD treatment had to reach the significance value to be included in the stepwise model. Separate sub-analysis of patients satisfying the 2010 ACR/EULAR criteria, and patients who were either RF-positive or anti-CCP–positive at baseline were also carried out.

To assess whether the pattern of HAQ scores over time differed between remission groups, a random effects model was used to allow for within-subject correlations. Differences in median HAQ, mean DAS28-CRP and relative frequency of patients in each of the DAS28-CRP categories between the three remission groups were analysed using a non-parametric test for trend (an extension of the Wilcoxon rank-sum test). All analyses were conducted using Stata V.13 (Stata Corp, College Station, TX, USA).

## Results

A total of 868 patients with complete joint count data were included in the primary analysis. Two-thirds (66%) were female, and the median symptom duration at baseline was 6.5 [Interquartile Range (IQR): 4.1–11.1] months (Table 1). Of these patients, 60% satisfied the 2010 ACR/EULAR criteria for RA. Comparison between these 868 patients and patients with missing joint count at ⩾1 follow-up visit (293 patients, including 29 patients who died during follow-up) showed statistically significant differences for, respectively: proportion of females (66 *vs* 59%, P = 0.03), number of swollen joints at baseline [median 3, IQR (1–7) *vs* median 2, IQR (0–7), P = 0.02], proportion of patients satisfying the 2010 ACR/EULAR criteria for RA at baseline (60 *vs* 47%, P < 0.001), proportion of patients receiving DMARD treatment at baseline (51 *vs* 43%, P = 0.009) and time between symptom onset and starting DMARDs [median 6.28, IQR (3.94–11.68) *vs* median 5.22, IQR (3.02–11.76) months, P = 0.04].

**Table 1:** Baseline characteristics

Characteristics	Whole cohort		Never remission		Intermittent remission		Sustained remission	
		n = 868		n = 471		n = 296		n = 101
Age at onset, years, mean (s.d.)	55.9 (14.6)	868	56.5 (14.1)	471	55.1 (14.9)	296	56.0 (15.9)	101
Symptom duration, months, median (IQR)	6.5 (4.1–11.1)	868	6.7 (4.4–11.6)	471	6.3 (3.9–11.0)	296	5.5 (3.8–10.1)	101
Female, %	65.8	571/868	75.2	354/471	58.1	172/296	44.6	45/101
Swollen joints, 51 count, median (IQR)	3 (1–7)	868	5 (2–9)	471	2 (0–5)	296	2 (0–5)	101
Tender joints, 51 count, median (IQR)	4 (1–13)	868	8 (3–18)	471	2 (0–8)	296	1 (0–3)	101
CRP, median (IQR), mg/l	11.2 (4.9–22.0)	734	11.0 (4.1–23.1)	401	11.8 (6.0–21.7)	245	11.3 (4.4–21.7)	88
RF-positive, %	42.5	343/807	43.1	188/436	44.8	124/277	33.0	31/94
Anti-CCP–positive, %	33.5	241/720	33.9	131/386	37.0	90/243	22.0	20/91
DAS28, mean (s.d.)	3.8 (1.3)	734	4.1 (1.3)	401	3.4 (1.2)	245	3.1 (1.1)	88
HAQ, median (IQR)	0.9 (0.4–1.5)	853	1.3 (0.6–1.8)	462	0.6 (0.3–1.1)	292	0.5 (0.0–1.0)	99
Satisfied 2010 ACR/EULAR criteria for RA, %	59.8	519/868	69.9	329/471	51.0	151/296	38.6	39/101
Taking DMARDS, %	51.4	446/868	51.0	240/471	54.4	161/296	44.6	45/101
Time between symptom onset and starting DMARDS, months, median (IQR)	6.3 (3.9–11.7)	684	6.6 (4.0–12.6)	384	6.2 (3.5–11.0)	237	5.2 (3.0–10.1)	63
Starting DMARD treatment within 3 months of symptom onset, %	17.1	117/684	13.5	52/384	21.9	52/237	20.6	13/63
Taking oral steroids, %	24.9	216/868	25.1	118/471	24.7	73/296	24.8	25/101
Never smoked, %	39.8	339/852	39.6	185/467	41.1	117/285	37.0	37/100
Current smoker, %	21.5	183/852	22.1	103/467	19.7	56/285	24.0	24/100
Smoked in the past, %	38.7	330/852	38.3	179/467	39.3	112/285	39.0	39/100
Obese (BMI >30 kg/m^2^), %	26.3	224/853	32.3	149/462	21.2	62/292	13.1	13/99
At least one comorbidity,^a^ %	67.9	589/868	73.3	345/471	64.9	192/296	51.5	52/101
Hypertensive	39.4	342/868	43.3	204/471	37.5	111/296	26.7	27/101
Depressed	36.2	314/868	42.5	200/471	31.8	94/296	19.8	20/101

^a^At least one of the following comorbidities: angina, hypertension, heart attack, heart failure, stroke, transient ischaemic attack, diabetes, stomach ulcer, liver disease, kidney failure, cancer (except skin cancer), psoriasis, depression, glaucoma.

The numbers of patients in the NR, IR and SR groups were 471 (54%), 296 (34%) and 101 (12%), respectively (Table 1). In univariate ordinal regression analysis, female sex was strongly associated with being in a lower remission group (i.e. less likely to achieve remission) (Table 2). Age at onset was not a predictor. A higher number of swollen and tender joints, higher DAS28-CRP, higher HAQ and satisfying 2010 ACR/EULAR criteria for RA and having at least one comorbidity at baseline were also significantly associated with being in a lower remission group. Of the individual comorbidities considered, hypertension (odds ratio, OR = 0.67, 95% CI: 0.51, 0.87) and depression (OR = 0.52, 95% CI: 0.40, 0.69) were associated with being in a lower remission group in univariate analysis. Obesity, although not considered as a comorbidity in the context of this study, was also associated with reduced odds of being in a higher remission category (OR = 0.49, 95% CI: 0.36, 0.66). These conditions were independently associated with remission group when we added all three to a multivariate model adjusted for age and gender (data not shown). Among patients who were receiving DMARD treatment at baseline, the time from symptom onset to starting DMARD treatment, modelled as a continuous variable, was not significantly associated with remission group in univariate ordinal regression analysis. However, patients who started DMARD treatment within 3 months of symptom onset (n = 117) were more likely to be in a higher remission group (i.e. more likely to achieve remission) (OR = 1.66, 95% CI: 1.14, 2.42). These results remained significant after adjusting for age, sex and steroid use at baseline. Adjusting additionally for DMARD use at baseline did not materially alter the results (data not shown). Similar results were seen in sub-analyses of patients satisfying the 2010 ACR/EULAR criteria (Table 3), and in patients who were RF- and/or anti-CCP–positive (supplementary Table S1, available at *Rheumatology* Online).

**Table 2:** Baseline predictors of remission group using ordinal logistic regression

Characteristics	Model 1^a^		Model 2^b^		Model 3^c^	
	OR (95% CI)	P-value	OR (95% CI)	P-value	OR (95% CI)	P-value
Age at onset, per year	1.00 (0.99, 1.00)	0.3	–	–	–	–
Symptom duration, per month	0.98 (0.96, 1.00)	0.09	0.98 (0.95, 1.00)	0.06	–	–
Female *vs* male	0.39 (0.30, 0.51)	<0.001	–	–	0.47 (0.35, 0.63)	<0.001
Swollen joints, per joint	0.94 (0.92, 0.96)	<0.001	0.94 (0.92, 0.97)	<0.001	–	–
Tender joints, per joint	0.92 (0.90, 0.94)	<0.001	0.92 (0.91, 0.94)	<0.001	0.94 (0.93, 0.96)	<0.001
CRP per mg/l	1.00 (0.99, 1.01)	1.0	1.00 (0.99, 1.01)	0.83	–	–
RF-positive, yes *vs* no	0.88 (0.67, 1.16)	0.37	0.90 (0.68, 1.19)	0.46	–	–
Anti-CCP–positive, yes *vs* no^d^	0.86 (0.64, 1.16)	0.33	0.92 (0.68, 1.25)	0.6	–	–
Never remission	Referent	Referent	Referent	Referent	–	–
Intermittent remission	1.15 (0.82, 1.60)	0.43	1.22 (0.86, 1.72)	0.26	–	–
Sustained remission	0.55 (0.32, 0.94)	0.03	0.60 (0.34, 1.04)	0.07	–	–
DAS28, per unit	0.57 (0.50, 0.65)	<0.001	0.59 (0.52, 0.67)	<0.001	–	–
HAQ, per unit	0.39 (0.32, 0.47)	<0.001	0.42 (0.34, 0.51)	<0.001	0.59 (0.48, 0.74)	<0.001
Satisfied 2010 ACR/EULAR criteria for RA, yes *vs* no	0.39 (0.30, 0.51)	<0.001	0.42 (0.32, 0.55)	<0.001	–	–
Time between symptom onset and starting DMARDS, per month	0.99 (0.98, 1.00)	0.17	0.99 (0.98, 1.00)	0.14	–	–
Starting DMARD treatment within 3 months of symptom onset, yes *vs* no	1.66 (1.14, 2.43)	0.008	1.64 (1.11, 2.41)	0.012	–	–
Never smoked	referent	referent	referent	referent	–	–
Current smoker	0.99 (0.83, 1.18)	0.91	0.88 (0.73, 1.06)	0.17	–	–
Smoked in the past	1.03 (0.77, 1.38)	0.86	0.88 (0.65, 1.20)	0.44	–	–
Obese, BMI>30 kg/m^2^, yes *vs* no	0.49 (0.36, 0.66)	<0.001	0.49 (0.36, 0.68)	<0.001	0.70 (0.50, 0.99)	0.04
At least one comorbidity,^e^ yes *vs* no	0.55 (0.42, 0.73)	<0.001	0.57 (0.43, 0.76)	<0.001	–	–
Hypertensive	0.67 (0.51, 0.87)	0.003	0.63 (0.47, 0.84)	0.002	0.67 (0.50, 0.90)	0.008
Depressed	0.52 (0.40, 0.69)	<0.001	0.57 (0.43, 0.76)	<0.001	0.74 (0.55, 1.00)	0.05

^a^Model 1 is univariate analysis.

^b^Model 2 is adjusted for age, sex and steroid use at baseline.

^c^Model 3 includes covariates selected by a stepwise procedure. A significance value of <0.1 was used as the cut-off to be included in the model.

^d^Multinomial regression analysis was used for anti-CCP, since this variable did not satisfy the proportional odds assumption of ordinal regression.

^e^At least one of the following comorbidities: angina, hypertension, heart attack, heart failure, stroke, transient ischaemic attack, diabetes, stomach ulcer, liver disease, kidney failure, cancer (except skin cancer), psoriasis, depression, glaucoma.

**Table 3:** Baseline predictors of remission group for patients satisfying the 2010 ACR/EULAR criteria for RA at baseline

Characteristics	Model 1^a^		Model 2^b^		Model 3^c^	
	OR (95% CI)	P-value	OR (95% CI)	P-value	OR (95% CI)	P-value
Age at onset, per year	1.00 (0.99, 1.01)	0.9	–	–	0.99 (0.97, 1.00)	0.09
Symptom duration, per month	1.00 (0.97, 1.04)	0.85	1.00 (0.96, 1.03)	0.88	–	–
Female *vs* male	0.33 (0.22, 0.48)	<0.001	–	–	0.36 (0.24, 0.54)	<0.001
Swollen joints, per joint	0.97 (0.94, 1.00)	0.02	0.97 (0.94, 0.99)	0.02	–	–
Tender joints, per joint	0.94 (0.92, 0.95)	<0.001	0.94 (0.92, 0.95)	<0.001	0.95 (0.93, 0.97)	<0.001
CRP, per mg/l	1.00 (1.00, 1.01)	0.59	1.00 (1.00, 1.01)	0.49	–	–
RF-positive, yes *vs* no	1.66 (1.13, 2.42)	0.009	1.56 (1.06, 2.31)	0.02	–	–
Anti-CCP–positive, yes *vs* no^d^						
Never remission	Referent	Referent	Referent	Referent	–	–
Intermittent remission	2.12 (1.37, 3.28)	0.001	2.16 (1.38, 3.39)	0.001	–	–
Sustained remission	1.26 (0.62, 2.54)	0.5	1.28 (0.62, 2.65)	0.5	–	–
DAS28, per unit	0.58 (0.49, 0.70)	<0.001	0.60 (0.50, 0.72)	<0.001	–	–
HAQ, per unit	0.41 (0.31, 0.54)	<0.001	0.45 (0.34, 0.59)	<0.001	0.63 (0.46, 0.84)	0.002
Time between symptom onset and starting DMARDS, per month)	0.99 (0.97, 1.01)	0.22	0.99 (0.97, 1.01)	0.28	–	–
Starting DMARD treatment within 3 months of symptom onset, yes *vs* no	1.59 (0.98, 2.58)	0.06	1.62 (0.99, 2.66)	0.06	–	–
Never smoked	Referent	Referent	Referent		–	–
Current smoker	1.24 (0.77, 2.00)	0.38	0.92 (0.55, 1.54)	0.76	–	–
Smoked in the past	1.27 (0.84, 1.90)	0.25	1.04 (0.67, 1.59)	0.87	–	–
Obese, BMI >30 kg/m^2^, yes *vs* no	0.52 (0.35, 0.79)	0.002	0.56 (0.37, 0.86)	0.009	–	–
At least one comorbidity,^e^ yes *vs* no	0.78 (0.53,1.15)	0.21	0.0.77 (0.52, 1.15)	0.21	–	–
Hypertensive	0.78 (0.54, 1.12)	0.18	0.72 (0.48, 1.06)	0.1	–	–
Depressed	0.64 (0.45, 0.92)	0.02	0.70 (0.48, 1.02)	0.07	–	–

^a^Model 1 is univariate analysis.

^b^Model 2 is adjusted for age, sex and steroid use at baseline.

^c^Model 3 includes covariates selected by a stepwise procedure. A significance value of <0.1 was used as the cut-off to be included in the model.

^d^Multinomial regression analysis was used for anti-CCP, since this variable did not satisfy the proportional odds assumption of ordinal regression.

^e^At least one of the following comorbidities: angina, hypertension, heart attack, heart failure, stroke, transient ischaemic attack, diabetes, stomach ulcer, liver disease, kidney failure, cancer (except skin cancer), psoriasis, depression, glaucoma.

In multivariate regression analysis, female sex (OR = 0.47, 95% CI: 0.35, 0.63), higher tender joint count (OR = 0.94, 95% CI: 0.93, 0.96), higher HAQ score (OR = 0.59, 95% CI: 0.48, 0.74), being obese (OR = 0.70, 95% CI: 0.50, 0.99), hypertensive (OR = 0.67, 95% CI: 0.50, 0.90) or depressed (OR = 0.74, 95% CI: 0.55, 1.00) at baseline were all independently associated with being in a lower remission group (i.e. less likely to achieve remission) (Table 2). Similar results were seen in a sub-analysis of patients satisfying the 2010 ACR/EULAR criteria (Table 3).

HAQ scores differed significantly between remission groups at baseline (Table 1) and over time (Table 4). Using a random effects model to allow for within-person correlation, we found that, after adjusting for age and sex, IR and SR were associated with a lower HAQ score over time compared with NR (β = −0.51, 95% CI: −0.60, −0.43 for IR, β = −0.85, 95% CI: −0.98, −0.72 for SR).

**Table 4:** HAQ scores over time and DAS28 score at fifth year anniversary

Characteristics	Never remission		Intermittent remission		Sustained remission		P^a^-value
		n = 471		n = 296		n = 101	
HAQ, median (IQR)							
First anniversary	1 (0.5–1.75)	467	0.5 (0–1)	293	0.125 (0–0.38)	101	<0.001
Second anniversary	1.25 (0.63–1.75)	467	0.375 (0.13–1)	293	0 (0–0.25)	100	<0.001
Third anniversary	1.125 (0.63–1.88)	469	0.38 (0–1)	294	0 (0–0.25)	100	<0.001
Fifth anniversary	1.25 (0.63–1.88)	469	0.38 (0–1)	287	0 (0–0.25)	100	<0.001
DAS28-CRP, mean (s.d.)							
Fifth anniversary	1.60 (0.94)	232	0.54 (0.78)	134	0.23 (0.55)	52	<0.001
DAS28-CRP category, fifth anniversary, %							
Remission, DAS28 <2.6	17.7	41/232	61.2	82/134	82.7	43/52	<0.001
Low disease activity, 2.6≤DAS28<3.2	19.4	45/232	25.4	34/134	11.5	6/52	
Moderate disease activity, 3.2≤DAS28<5.1	48.3	112/232	11.2	15/134	5.8	3/52	
High disease activity, DAS28≥5.1	14.7	34/232	2.2	3/134	0.0	0/52	

^a^P-value from non-parametric test for trend.

The mean (s.d.) DAS28-CRP score was significantly higher at year 5 in the NR group [1.60 (0.94)] than in the IR [0.54 (0.78)] or SR [0.23 (0.55)] groups (Table 4). Similarly, a much higher proportion of patients in the SR group were in DAS28-remission (83%) than in the IR (61%) or NR (18%) group. The fact that 18% of the NR group were in DAS28 remission at year 5 shows that our remission criteria are stricter than the DAS28 criteria.

## Discussion

In this observational study of patients with IP, we found that 46% of patients were in remission [defined as no swollen or tender joints (out of 51)] at one or more time-points during a 5-year follow-up period. Thirty-four percent of patients achieved IR and only 12% of patients achieved SR in that time frame. Female sex, higher tender joint counts, higher HAQ score and being hypertensive, depressed or obese at baseline were independent predictors of being in a lower remission group (i.e. of being less likely to achieve remission). Starting DMARD treatment within 3 months of symptom onset was associated with being in a higher remission group (i.e. being more likely to achieve remission), but only in univariate and age, sex and steroid use at baseline adjusted analyses.

Comparison of remission rates between studies is difficult because of differences in definitions of remission, the number of times remission was assessed, stage of disease, patient characteristics and treatment regimens between studies. The systematic review conducted Katchamart *et al.* [5] included papers reporting remission rates varying from 4 to 53%. Remission criteria proposed for RA include some based on thresholds of disease activity measures such as the DAS28 [22], the Simplified Disease Activity Index [23] or the Clinical Disease Activity Index [24]; some based on a long list of symptoms such as the strict preliminary criteria proposed by Pinals *et al.* [25] or Boolean logic such as the 2011 ACR/EULAR definition [26]. The choice of remission definition has a marked effect on the proportion of patients classified as being in remission [27–30]. For example, Kuriya *et al.* [29] found that the ACR/EULAR Boolean criteria and the DAS28 criteria for remission classified 18 and 40%, respectively, of patients with early RA as being in remission at 12 months follow-up.

In our cohort, 46% of patients achieved at least IR + SR during a 5-year follow-up. Only 12% of patients achieved persistent remission. Ellerby *et al.* [31] reported 44/295 (14.9%) patients achieved SR, defined as DAS28 < 2.6 at two consecutive time points, measured annually over a 5-year follow-up. Gossec *et al.* [32] reported 30/191 (15.7%) achieved SR, defined as DAS < 1.6 [32] at both third and fifth year anniversaries. In our cohort, only 5% of women achieved persistent remission, compared with 19% of men, which is in agreement with other studies reporting that women are less likely to achieve remission [13, 15, 33, 34]. The reasons why women are less likely to achieve remission are unclear, although sex hormones may play a role in the pathophysiology of RA, with androgens anti-inflammatory and oestrogens immune response enhancers at physiological concentrations [34]. It is also possible that women may be more likely to have co-existent primary general OA, contributing to a higher tender joint count.

Patients who were obese at baseline were less likely to achieve remission, independent of other comorbidities. Other studies have reported a similar negative association between obesity and remission and low disease activity [31, 35, 36]. With the prevalence of obesity rising globally [37], this is likely to become an increasingly important factor in determining outcome for patients. Adipose tissue functions as an endocrine organ, releasing pro-inflammatory and anti-inflammatory adipokines, which could play a role in sustaining joint inflammation and raised inflammatory markers [38, 39].

We found that patients in the IR and NR group were more likely to be depressed at baseline. There is prior evidence of a relationship between inflammation and depression [40, 41], with a suggestion that depression may precede inflammation [42, 43]. While we cannot be sure that depressive symptoms preceded disease symptoms at baseline in our cohort, it is possible that pre-existing depressive symptoms may influence disease progression. There is a bi-directional relationship between functional disability and depression in patients with chronic medical illness [44]. Functional disability predicts the development of depression, and depressive symptoms are a risk factor for the progression of disability. Additionally, depression has been linked to adverse health-risk behaviour such as smoking, overeating and non-compliance with recommended treatment.

Strengths of our study include a large cohort of patients with early IP, with detailed clinical, treatment, demographic and comorbidity data. Having a 5-year follow-up allowed us to identify baseline factors associated with IR and SR, rather than factors associated with remission at a single point in time. Limitations of our study include our definition of remission, which does not include a blood marker of inflammation. We were not able to include a blood marker of inflammation in our definition of remission because blood was collected only at the baseline and fifth year assessments. This could result in some misclassification of patients who are in remission of their inflammatory arthritis but have one or more tender joints due to other conditions such as FM. However, the definition of remission we have used is strict and, unlike the DAS-28 definition of remission, does not allow for any swollen or tender joints [45]. Our findings that women and patients with a higher HAQ score at baseline were less likely to achieve remission is in agreement with other studies using a DAS28 definition of remission [46, 47]. It is known that patients with RA can have altered body composition, with a decrease in lean muscle mass. Other methods of determining obesity, such as hip to waist ratio, bioimpedence, or whole-body MRI may be more appropriate for patients with RA [48], though these measurements were not taken in our study. Another limitation of our study is that comorbidities were self-reported.

## Conclusions

The current aim of the treatment of IP and RA is to achieve early and sustained remission. However, in our study we found that only about 12% of patients with IP achieved sustained remission (by our definition) during a 5-year follow-up. Sex, number of tender joints, HAQ score and being obese, hypertensive or depressed at baseline were all independently associated with the remission group. These factors may be useful in predicting which patients are likely to achieve remission and, thus, in guiding treatment choice and timing.

## Supplementary Material

Supplementary Data
