# Supplementary material for: Predictors and outcomes of sustained, intermittent or never achieving remission in patients with recent onset inflammatory polyarthritis: results from the Norfolk Arthritis Register
Source: Rheumatology (Oxford). 2016 May 24;55(9):1601–9. doi: 10.1093/rheumatology/kew210 (PMC4993956; doi:10.1093/rheumatology/kew210)
Supplement: Supplementary Data [file supp_55_9_1601__index.html]

Predictors and outcomes of sustained, intermittent or never achieving remission in patients with recent onset inflammatory polyarthritis: results from the Norfolk Arthritis Register — Predictors and outcomes of sustained, intermittent or never achieving remission in patients with recent onset inflammatory polyarthritis: results from the Norfolk Arthritis Register — Supplementary Data 

# Predictors and outcomes of sustained, intermittent or never achieving remission in patients with recent onset inflammatory polyarthritis: results from the Norfolk Arthritis Register

## Supplementary Data

files

- Supplementary Data - docx file
