# Supplementary material for: Predictors and outcomes of sustained, intermittent or never achieving remission in patients with recent onset inflammatory polyarthritis: results from the Norfolk Arthritis Register
Source: Rheumatology (Oxford). 2016 May 24;55(9):1601–9. doi: 10.1093/rheumatology/kew210 (PMC4993956; doi:10.1093/rheumatology/kew210)
Supplement: Supplementary Data [file supp_kew210_rhe-15-1664-File003.docx]

**Supplementary Figure S1. Number of patients included in the primary analysis**

**1516**  Patients recruited to NOAR from 2000 to 2008

**352**  Patients excluded with symptom duration ≥2 years at baseline

**1164**

**29** Patients excluded who died during follow up

**1135**

**267** Patients excluded who had joint count data missing at ≥1 follow up visit

**868** Patients included in the primary analysis

**Supplementary Table S1.** Baseline predictors of remission group for patients who are RF positive or anti-CCP positive at baseline

|  | **Model 1^a^** | | **Model 2^b^** | | **Model 3^c^** | |
| --- | --- | --- | --- | --- | --- | --- |
|  | **OR (95% CI)** | *p* | **OR (95% CI)** | *p* | **OR (95% CI)** | *p* |
| Age at onset, per year | 1 .00(0.98, 1.01) | 0.7 | - | - | - | - |
| Symptom duration, per month | 1.01 (0.97, 1.05) | 0.6 | 1.00 (0.96, 1.03) | 0.9 | - | - |
| Female vs male | 0.35 (0.23, 0.53) | <0.001 | - | - | 0.32 (0.19, 0.53) | <0.001 |
| Swollen joints, per joint | 0.94 (0.91, 0.97) | <0.001 | 0.94 (0.91, 0.97) | 0.001 | - | - |
| Tender joints, per joint | 0.92 (0.89, 0.94) | <0.001 | 0.92 (0.89, 0.94) | <0.001 | 0.94 (0.91, 0.97) | <0.001 |
| CRP, per mg/l | 1.00 (0.99, 1.01) | 0.9 | 1.00 (0.99, 1.01) | 0.9 | - | - |
| RF-positive, yes vs no | - |  | - | - | - | - |
| Anti-CCP positive, yes vs no | 0.98 (0.63, 1.52) | 0.9 | - | - | - | - |
| DAS28, per unit | 0.60 (0.51, 0.72) | <0.001 | 0.61 (0.51, 0.72) | <0.001 | - | - |
| HAQ, per unit | 0.47 (0.35, 0.61) | <0.001 | 0.50 (0.38, 0.67) | <0.001 | 0.71 (0.50, 1.01) | 0.06 |
| Satisfied 2010 ACR/EULAR criteria for RA, yes vs no |  |  | 0.47 (0.28, 0.79) | 0.004 | - | - |
| Time between symptom onset and starting DMARDS, per month | 1.00 (0.98, 1.02) | 1.0 | 1.00 (0.98, 1.02) | 0.8 | - | - |
| Starting DMARD treatment within 3 months of symptom onset, yes vs no | 1.93 (1.15, 3.23) | 0.01 | 1.88 (1.11, 3.19) | 0.02 | - | - |
| Never smoked | referent |  | referent |  | - | - |
| Current smoker | 1.33 (0.86, 2.06) | 0.2 | 1.09 (0.65, 1.81) | 0.8 | - | - |
| Smoked in the past | 1.38 (0.84, 2.26) | 0.2 | 1.13 (0.72, 1.78) | 0.6 | - | - |
| Obese, BMI>30kg/m^2^, yes vs no | 0.58 (0.36, 0.95) | 0.03 | 0.6 (0.36, 1) | 0.05 | - | - |
| At least one comorbidity^d^, yes vs no | 0.61 (0.40, 0.91) | 0.02 | 0.61 (0.4, 0.93) | 0.02 | - | - |
| Hypertensive | 0.64 (0.43, 0.95) | 0.03 | 0.59 (0.39, 0.91) | 0.02 | 0.62 (0.37, 1.03) | 0.07 |
| Depressed | 0.66 (0.44, 0.97) | 0.04 | 0.71 (0.48, 1.06) | 0.1 | - | - |

^a^Model 1 is univariate analysis. ^b^Model 2 is adjusted for age, sex and steroid use at baseline. ^c^Model 3 includes covariates selected by a stepwise procedure. A significance value of <0.1 was used as the cut-off to be included in the model. ^d^At least one of the following comorbidities: angina, hypertension, heart attack, heart failure, stroke, transient ischaemic attack, diabetes, stomach ulcer, liver disease, kidney failure, cancer (except skin cancer), psoriasis, depression, glaucoma. CRP: C-reactive protein (CRP); RF: rheumatoid factor; anti-CCP: anti-cyclic citrullinated
